# Spatial and Temporal Control of Transgene Expression in Zebrafish

**DOI:** 10.1371/journal.pone.0092217

**Published:** 2014-03-18

**Authors:** Alexander A. Akerberg, Scott Stewart, Kryn Stankunas

**Affiliations:** 1 Institute of Molecular Biology, University of Oregon, Eugene, Oregon, United States of America; 2 Department of Biology, University of Oregon, Eugene, Oregon, United States of America; University of North Carolina at Chapel Hill, United States of America

## Abstract

Transgenic zebrafish research has provided valuable insights into gene functions and cell behaviors directing vertebrate development, physiology, and disease models. Most approaches use constitutive transgene expression and therefore do not provide control over the timing or levels of transgene induction. We describe an inducible gene expression system that uses new tissue-specific zebrafish transgenic lines that express the Gal4 transcription factor fused to the estrogen-binding domain of the human estrogen receptor. We show these Gal4-ERT driver lines confer rapid, tissue-specific induction of UAS-controlled transgenes following tamoxifen exposure in both embryos and adult fish. We demonstrate how this technology can be used to define developmental windows of gene function by spatiotemporal-controlled expression of constitutively active Notch1 in embryos. Given the array of existing UAS lines, the modular nature of this system will enable many previously intractable zebrafish experiments.

## Background

Research using transgenic zebrafish lines has greatly contributed to our understanding of vertebrate biology. Transgenic zebrafish are used widely for both gain and loss of function experiments as well as a means to track specific cell populations. All such studies require careful consideration regarding the location, timing, and levels of transgene expression. For instance, although constitutive ubiquitous promoters generally produce high levels of transgene expression and generate robust phenotypes, they do not differentiate cell-type specificity or the timing of gene function. To overcome these limitations, tissue specific promoters are used to direct transgene expression to discrete cell lineages and tissue types. Temporal control of transgene expression further allows the determination of windows during which a gene functions, a feature particularly useful for developmental biology research. Additionally, multiple roles for a gene during development can be distinguished by timing the induction of an appropriate transgene. Temporal control of transgene expression in zebrafish is typically achieved using heat-shock sensitive promoters [1], although small molecule-controlled inducible promoters also can control the timing and tune levels of transgene expression [2].

One elegant method for transgene expression in metazoans uses the Gal4/UAS two component transcriptional activation switch from *Saccharomyces cerevisiae* [3], [4]. Gal4 is a transcription factor that functions in yeast galactose metabolism [5] and binds to a unique DNA sequence found between the GAL1 and GAL10 genes [6]. A version of this sequence is now commonly referred to as a UAS (for Upstream Activating Sequence). A discrete DNA-binding domain (DBD) of Gal4 is both necessary and sufficient for high-specificity binding to a UAS [7]. Gal4 can activate transcription in heterologous systems by using synthetic promoters consisting of basal transcription initiating elements combined with tandem repeats of the UAS sequence [3], [4], [8]–[10]. Modified Gal4-based chimeric proteins comprising the Gal4 DBD fused to the strong transcriptional activation domain from the viral VP16 protein are particularly potent and specific transcriptional activators [11], [12]. The Gal4/UAS system can be readily adaptable for transgenesis studies in diverse biological systems given that any gene of interest can be inserted downstream of a UAS/minimal promoter cassette.

In the Gal4/UAS system, the spatial domain of UAS-controlled transgene expression is determined by the promoter used to express Gal4 [13]. Additional refinements of the Gal4/UAS system provide temporal control of transgene expression. Taking advantage of the modular nature of Gal4 [7] and steroid hormone receptors [14], chimeric proteins have been produced that fuse the hormone binding domain from either the estrogen (ER) or glucocorticoid receptor (GR) to Gal4 [14]. The resulting Gal4-ER and Gal4-GR fusion proteins activate UAS-controlled reporter genes only in the presence of the cognate steroid hormone [11], [14]. To overcome effects of endogenous steroid hormones on Gal4-ER chimeras, an ER variant (known as ERT) with reduced affinity for naturally occurring estradiol but very high affinity for the estradiol analogs tamoxifen and 4-hydroxy tamoxifen (4-OHT) has been developed [15], [16]. The collective features of Gal4-ERT-VP16 fusion proteins make them a potent, highly specific, and tightly controllable tool for transgene expression upon administration of either tamoxifen or 4-OHT [11], [12], [14]–[16].

The Gal4/UAS system has been adapted for use in zebrafish [17]–[19] and a wide-range of UAS lines are available that express, for example, wild-type or mutant proteins, fluorescent markers, and Cre recombinase for lineage-tracing studies. However, a search of available zebrafish lines from the Zebrafish International Resource Center (ZIRC) yields only a handful of Gal4 lines that use unique, well-characterized promoters. Further, the majority of available Gal4 lines lack the ability to control the timing of gene expression. This is particularly problematic for larval or adult studies when phenotypes arising from earlier transgene expression obscure or exclude the later studies. Inducible Gal4/UAS switches in zebrafish designed to overcome this problem rely on heat shock promoter control of Gal4 expression [1]. This approach produces strong expression but suffers from several major disadvantages, including a lack of cell-type specificity, poor control of expression kinetics, and spurious effects of repeated heat shocks.

To overcome these limitations, we adapted the tamoxifen controlled Gal4-ERT system for use in zebrafish. We describe the generation and characterization of three tissue specific tamoxifen-dependent Gal4-ERT driver lines. These lines achieve rapid, dose-dependent expression of UAS-controlled transgenes by simply adding tamoxifen or 4-OHT to zebrafish water. We also validate this approach for functional analysis by demonstrating that tamoxifen dependent, tissue-specific expression of a constitutively active intracellular Notch domain (NICD) produces dramatic defects in notochord development. This effect requires NICD expression during an early developmental window prior to 10 hpf. The tamoxifen-dependent Gal4-ERT system affords zebrafish researchers the ability to address many biological questions previously limited by reliance upon ubiquitous and/or non-inducible transgene expression.

## Materials and Methods

### Zebrafish

This study was carried out in strict accordance with the recommendations in the Guide for the Care and Use of Laboratory Animals (National Academies Press) and all steps were taken to minimize animal discomfort. Zebrafish were euthanized by overdose with Tricaine. The University of Oregon Institutional Animal Care and Use Committee (IACUC) approved all protocols. PHS assurance number for animal research: A-3009-01. The following established lines were used in this study: wild-type AB, *Tg(5xUAS:EGFP)zf82* [20], *Tg(14xUAS:LOX2272-LOXP-RFP-LOX2272-CFP-LOXP-YFP)a130* (*UAS:Zebrabow*) [21], and *Tg(5xUAS-E1b:6xMYC-notch1a)kca3* [17].

### Transgene construction

The Gateway cloning system (Invitrogen, Carlsbad, CA) and Tol2 kit [22] was used to generate transgenes capable of tamoxifen-dependent expression. First, the coding sequence for the hybrid transcription factor, Gal4-ERT2-VP16 [11], [16] was inserted into ME-MCS to generate the middle element vector pME-Gal4-ERT-VP16. 5E vectors were constructed as follows: A region of the *krt5* promoter [23] at chr23:10,284,249–10,286,542 (Zv9/danRer7) that drives expression in the epidermis was amplified by PCR from zebrafish genomic DNA and cloned into the Gateway compatible vector p5E-MCS [22] to generate p5E-*krt5*; the p5E-dusp6 plasmid containing the FGF-responsive *dusp6* promoter [24] at chr25: 18,817,373–18,827,789 (Zv9/danRer7) has been described previously [25]; the semi-ubiquitous hybrid promoter, made up of the frog *ef1α* enhancer and the rabbit β-globin intron, from pT2AL200R150G [26] was inserted into p5E-MCS creating p5E-ef1α. Finally, we used Gateway Clonase LRII enzyme (Invitrogen, Carlsbad, CA) to recombine each of 5E-*krt5*, 5E-*eflα*, or 5E-*dusp6* promoter elements with ME-Gal4TVP16, 3E-*polyA* [22], and a modified Tol2 destination vector containing a *myl7*-ECFP cassette as a marker for transgenesis [25].

### Generation of transgenic animals

Plasmid DNA for each construct was co-injected with capped RNA coding for the Tc transposase into one cell stage AB embryos at a concentration of 25 ng/μl as described previously [25]. Animals displaying ECFP expression in the heart at 48 hpf were selected and reared to adulthood. Founders were identified by crossing to *Tg(5xUAS:EGFP)* animals, treating progeny with tamoxifen (3 μM, Sigma) at 8 hpf, and visualizing EGFP expression at 24–48 hpf. Founders with EGFP-positive progeny were then outcrossed to AB fish. The subsequent generation was screened for single transgenic insertions by again crossing to *Tg(5xUAS:EGFP)* fish, treating embryo progeny with tamoxifen, and scoring the clutch for EGFP expression. Stable lines were maintained as heterozygotes by outcrossing to AB fish and picking animals with ECFP expression in the heart. Following this approach, we established the following lines: *Tg(krt5:Gal4-ERT-VP16,myl7:ECFP)b1234*, *Tg(dusp6:Gal4-ERT-VP16,myl7:ECFP)b1235*, *Tg(eflα:Gal4-ERT-VP16,myl7:ECFP)b1236*.

### Induction of transgenes by tamoxifen

For studies using embryos, 4-hydroxy-tamoxifen (4-OHT, Sigma) was dissolved in ethanol and added directly to the fish water at the times and concentrations indicated in the figure legends. Control animals were treated with the same volume of ethanol. For adult studies, tamoxifen (Sigma) dissolved in DMSO or 4-OHT dissolved in ethanol was added to fish water at a final concentration of 1 μM for 1 hour, after which animals were transferred to fresh fish water. Control animals were treated in the same manner with the same volume of vehicle. Animals were subjected to this regimen 3 consecutive days prior to imaging and tissue harvesting.

### Immunostaining and imaging

**Figure 1:**
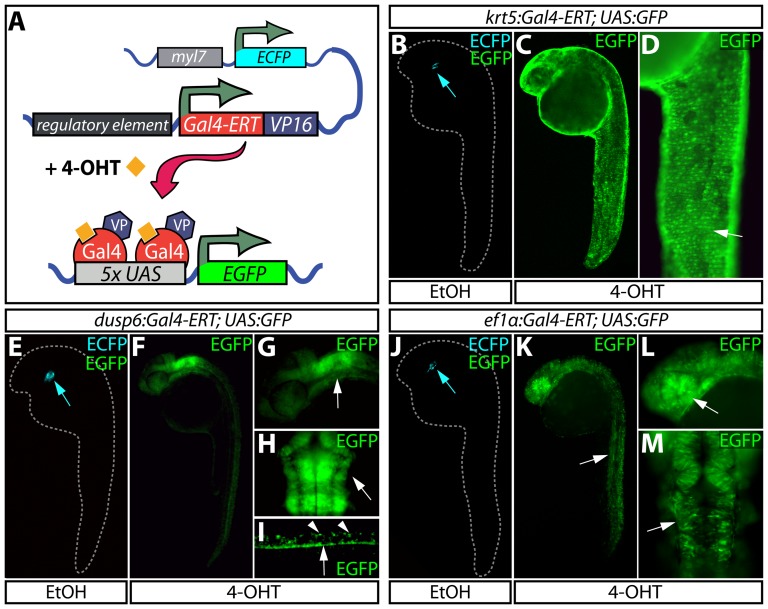
Temporally and spatially controlled transgene expression in zebrafish. (**A**) Schematic of transgenic constructs used in the Gal4-ERT system. A tamoxifen-responsive Gal4-ERT-VP16 construct is expressed from a tissue-specific promoter that activates any UAS-linked responder line (shown here as a *5xUAS:EGFP* reporter) upon tamoxifen or 4-OHT exposure (orange squares). The *myl7:ECFP* cassette serves as a transgenesis marker for the Gal4-ERT lines. (**B–D**) Visualization of EGFP expression in *Tg(krt5:Gal4-ERT-VP16; UAS:EGFP)* animals treated with ethanol (B) or 2 μM 4-OHT (C and D) from 4–24 hpf. The white arrow in panel D highlights expression of EGFP in the epidermis. (**E–I**) EGFP expression in *Tg(dusp6:Gal4-ERT-VP16; UAS:EGFP)* animals treated with vehicle (E) or 2 μM 4-OHT (F-I) from 4–24 hpf. In panels G and H, the white arrow indicates EGFP expression in the hindbrain and midbrain-hindbrain boundary. In panel I, arrowheads mark dorsal spinal cord neurons and the arrow points to EGFP expression in the floor plate. (**J–M**) Expression of EGFP in control (J) and 4-OHT treated (2 μM, K-M) *Tg(ef1α:Gal4-ERT-VP16; UAS:EGFP)* animals in a variety of cell types throughout the embryo including skeletal muscle (K, white arrow), the eye (L, white arrow), and the midbrain/midbrain-hindbrain boundary (M, white arrow). In panels B, E, and J, blue arrows point to myocardial ECFP expression, which represents the marker for transgenesis and serves as an internal control.

**Figure 2:**
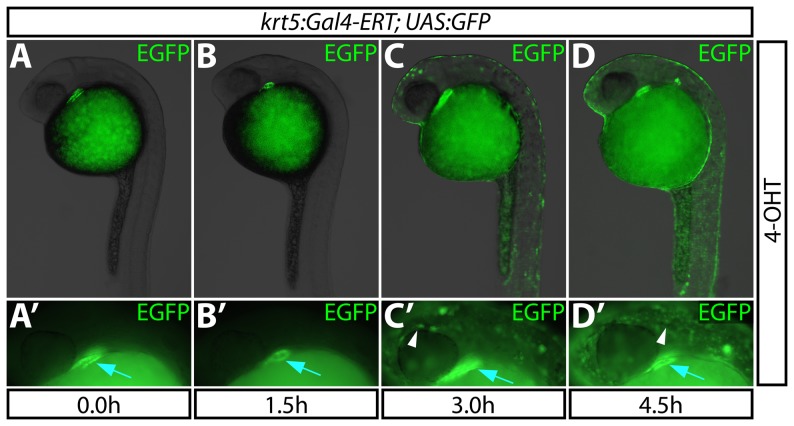
The Gal4-ERT system provides rapid induction of gene expression. (**A–D**) Kinetics of EGFP expression in *Tg(krt5:Gal4-ERT-VP16; UAS:EGFP)* animals upon administration of 2 μM 4-OHT at 24 hpf for 0–4.5 hours. (**A′–D′**) High magnification images demonstrating EGFP expression for each treatment. White arrowheads point to epidermal expression of EGFP; blue arrows indicate bleed-through from the heart muscle-specific ECFP transgenesis marker.

**Figure 3:**
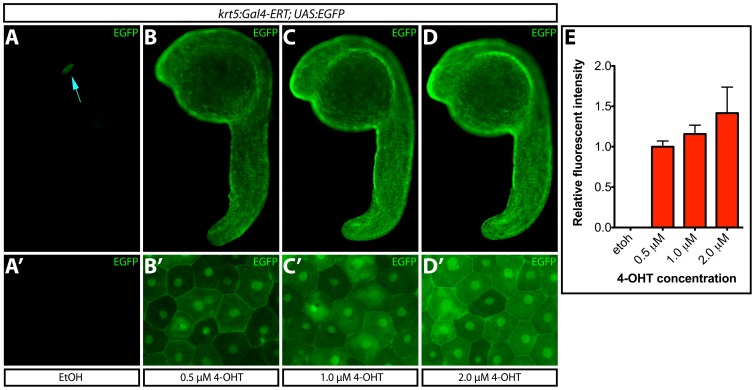
Transgene expression levels depend upon 4-OHT dosage. (**A–D**) EGFP expression upon treatment of *Tg(krt5:Gal4-ERT-VP16; UAS:EGFP)* zebrafish with ethanol or the indicated dose of 4-OHT from 4–24 hpf. The blue arrow indicates *myl7:ECFP* expression. (**A′–D′**) High-magnification images of ventral epidermis from fish in each treatment group. (**E**) Normalized EGFP intensity (to the 0.5 μM 4-OHT treated group) of fish treated with ethanol or 4-OHT. Error bars represent standard deviations.

For immunostaining embryos, animals were manually dechorionated and then fixed overnight at 4°C in phosphate-buffered saline (PBS) containing 4% paraformaldehyde. The next day, embryos were washed extensively in PBS containing 0.1% Tween-20 (PBST) then dehydrated though a methanol series. Embryos in 100% methanol were then transferred to −20°C for at least 24 h prior to rehydration to PBST. Embryos were incubated in blocking buffer (PBST containing 10% normal goat serum) for 1–2 h at room temperature with gentle mixing. Primary antibodies were sourced and diluted as follows in blocking buffer and then incubated overnight at 4°C: anti-GFP (1∶1000, Aves Labs), anti-myc epitope (1∶1000, Invitrogen). The next day embryos were washed 2×30′ in PBST followed by 2 h room temperature incubation in Alexa-conjugated secondary antibodies (Invitrogen) diluted 1∶1000 in blocking buffer. Embryos were then washed for 30′ in PBST, 30′ in PBS, mounted in low melt agarose, and imaged on a Nikon Eclipse Ti widefield inverted microscope. For Figures 1, 2, 3, and S2A, C, D–G, embryos were fixed as above, washed in PBS, mounted in low melt agarose, and the native fluorescence of marker proteins visualized by epifluorescent microscopy. For scoring phenotypes, animals were manually dechorionated and counted under a stereomicroscope. Adult animals were anesthetized with Tricaine and imaged under a Leica M165 FC stereomicroscope.

For paraffin-embedded eye sections, tamoxifen and control animals were sacrificed and eyes were immediately dissected and fixed overnight at 4°C in PBS containing 4% paraformaldehyde. The next day, samples were extensively washed in PBS, dehydrated, and embedded in paraffin. 7 μM sections were collected. Rehydrated sections were subjected to antigen retrieval by incubating in 0.25% trypsin for 20′ at 37°C, immunostaining with GFP and c-myc antibodies (described above), stained with Hoechst to label nuclei, mounted with Fluorogel (EMS), and imaged on a Leica DM4000B widefield microscope.

### Quantification of EGFP expression

High magnification images of ventral EGFP-labeled epidermal cells were captured with a Nikon Eclipse Ti widefield inverted microscope. Unprocessed images from four representative animals for each tamoxifen dose were then analyzed using the ImageJ (NIH) software package to generate pixel intensity values based on the strength of the fluorescent signal. Averaged pixel intensity values for each treatment were then normalized to that of the lowest tamoxifen dose (0.5 μM) and plotted as “relative fluorescent intensity”.

### In situ hybridization

In situ hybridizations were performed essentially as described by Thisse and Thisse [27]. Stained embryos were dehydrated in a methanol series and stored overnight at −20°C. Embryos were then rehydrated into PBST and transferred to 100% glycerol. Equilibrated embryos were mounted in glycerol and imaged using Rottermann contrast optics on a Leica M165 FC stereomicroscope.

## Results and Discussion

### Novel transgenic lines confer tamoxifen-dependent gene expression

We combined features of the widely used Gal4/UAS and ER-tamoxifen systems and adapted them for use in transgenic zebrafish (Figure 1A). As a first step, we established transgenic zebrafish expressing the tamoxifen-dependent Gal4-ERT transcription factor [11], [12], [14]–[16] in various tissues and cell types. To generate a broadly expressing Gal4-ERT line for temporal studies, we used a 1.1 kb region from the *Xenopus laevis elongation factor 1 alpha* (*ef1α*) promoter and rabbit β-globin intron sequence [26]. Additionally, we made a Gal4-ERT line driven by a 2.3 kb promoter region from the *keratin 5* (*krt5*) gene that has been reported to express in the epidermis exclusively [28]. Finally, we produced a line expressing Gal4 under the control of a 10 kb element from the *dual specificity phosphatase 6* (*dusp6*) gene. *Dusp6* (also known as *Mkp3*) is an FGF signaling responsive gene that is active in a variety of cells depending on the developmental stage and is induced in regenerating fins [24], [25], [29].

We crossed each of these driver lines to a *Tg(UAS:EGFP)* reporter line [20] and treated embryos with 2 μM 4-hydroxytamoxifen (4-OHT) from 4–24 hours post fertilization (hpf). In each case, 4-OHT treatment elicited distinct EGFP expression patterns in 25% of the fish, as expected from heterozygote intercrosses. *Tg(krt5:Gal4-ERT-VP16; UAS-EGFP)* fish exhibited strong signal solely in the epidermis (Figure 1B–D), mimicking the endogenous *krt5* mRNA expression pattern [28] (Figure S1). The *Tg(krt5:Gal4-ERT-VP16)* line was equally effective at producing a robust response when paired with additional UAS reporter lines (Figure S2A–C). *Tg(dusp6:Gal4-ERT-VP16; UAS-EGFP)* fish expressed EGFP in the expected pattern in the hindbrain, midbrain-hindbrain boundary, pharyngeal endoderm, notochord, and floor plate along with select dorsal motor neurons (Figure 1E–I) [29]. Although not ubiquitously expressed, the *Tg*(*ef1α:Gal4-ERT-VP16; UAS-EGFP)* line displayed EGFP expression in a wide variety of cell types throughout the embryo including those of the hindbrain, midbrain-hindbrain boundary, skeletal muscle and retina (Figure 1J–M). Unfortunately, the *Tg*(*ef1α:Gal4-ERT-VP16)* line becomes silenced prior to 48 hpf and is therefore only useful for early embryonic studies. In the absence of 4-OHT, no *UAS-EGFP* expression was detected with any of the Gal4-ERT lines; only the cardiac-specific *myl7:ECFP* which we employed as a transgenesis marker was observed (Figure 1B, 1E, and 1J). Additionally, we saw no indication of 4-OHT toxicity with any of the treatments. These results demonstrate that each of the Gal4-ERT lines provide the expected tissue specificity and strictly require 4-OHT for UAS-dependent transgene expression.

### The Gal4-ERT system provides rapid induction of gene expression

We next sought to determine the temporal resolution of the Gal4-ERT system by measuring how rapidly it can drive expression of *UAS:EGFP* in the presence of estradiol analogs. We placed 24 hpf *Tg(krt5:Gal4-ERT-VP16; UAS:EGFP)* embryos into fish-water containing 2 μM 4-OHT and monitored the emergence of EGFP expression. EGFP was first detected in the epidermis three hours post treatment. However, the expression was faint and non-homogenous (Figure 2C and 2C′). By four and a half hours post 4-OHT exposure, EGFP expression was markedly more robust and labeled nearly the entire epidermis (Figure 2D and 2D′). These results indicate that the Gal4-ERT system possesses activation kinetics suitable for time-sensitive studies of rapidly developing zebrafish embryos.

### Transgene expression levels can be varied by tamoxifen dosage

In addition to spatiotemporal control over transgene expression, it is often desirable to control levels of transgene expression. Therefore, we determined if varying the dose of 4-OHT would affect the degree of transgene expression. We treated *Tg(krt5:Gal4-ERT-VP16; UAS:EGFP)* embryos at sphere-stage with various concentrations of 4-OHT. At 24 hpf, embryos were harvested and fixed for imaging. We determined the fraction of EGFP-expressing epidermal cells and measured their normalized EGFP intensity. 4-OHT concentrations of 0.25 μM failed to produce detectable EGFP expression (data not shown). Embryos treated with 0.5 μM 4-OHT exhibited low-level EGFP in all presumptive epidermal cells (Figure 3A-B). Increasing the 4-OHT dose to 1 μM or 2 μM produced a relative gain in EGFP signal intensity with no indication of toxic effects (Figure 3C–E). Overall, increasing the dose from 0.5 μM to 2 μM yielded a 28% increase in EGFP levels (p<0.05). At all effective concentrations, EGFP levels differed from cell-to-cell, which may reflect varying EGFP accumulation depending on cell cycle stage and the time lapse since a given epidermal cell was established and initiated *krt5* promoter activity. We repeated the experiment using *Tg(krt5:Gal4-ERT-VP16; UAS:Zebrabow)* fish, which expressed mCherry in the majority of epidermal cells when exposed to 0.5 μM 4-OHT (Figure S2D–E). Treating animals with 1 or 2 μM 4-OHT again produced notably higher mCherry expression levels and no sign of toxic effects (Figure S2F–G). Together, these results indicate that the Gal4-ERT system can be used to tune transgene expression to a desired level by titrating the concentration of 4-OHT.

### Inducible transgene expression in adult zebrafish

**Figure 4:**
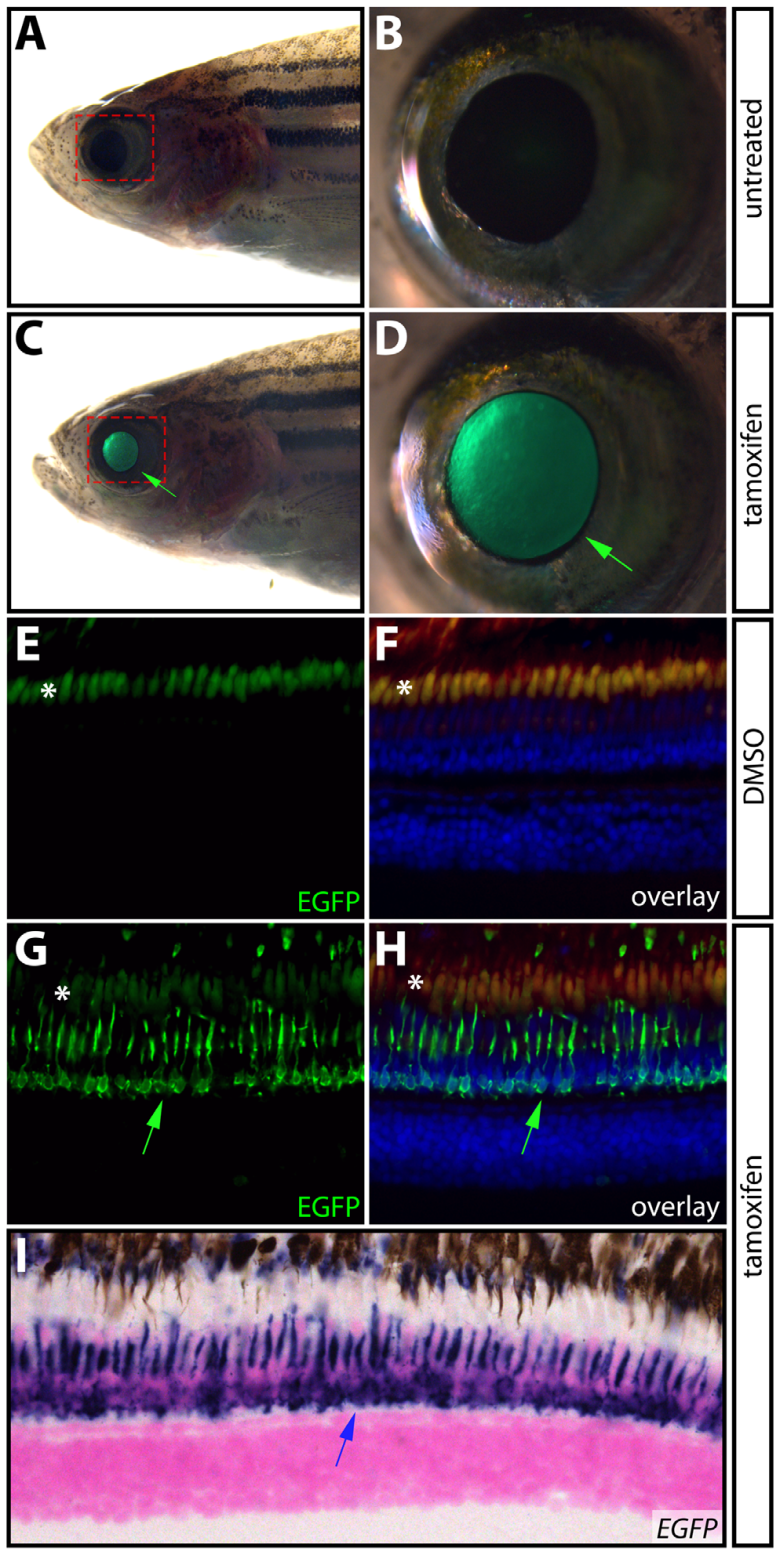
Inducible transgene expression in adult zebrafish. (**A–D**) Expression of EGFP in the eye of a single adult *Tg(krt5:Gal4-ERT-VP16; UAS:EGFP)* zebrafish prior to (A and B) and after (C and D) treatment with 1 μM tamoxifen for one hour per day for three consecutive days. Green arrows point to EGFP expression in the eye. The areas bounded by the dashed red box in A and C are shown at high magnification in B and D, respectively. (**E–H**) Immunostaining of paraffin sections with anti-EGFP antibodies (shown in green) in eyes from DMSO- (E and F) and tamoxifen-treated (G and H) *Tg(krt5:Gal4-ERT-VP16; UAS:EGFP)* fish. Panels F and H show overlays with anti-EGFP antibody staining in green, Hoechst-stained nuclei in blue, and auto-fluorescence in red. Fish were drug treated as in A–D. Green arrows indicate EGFP expression in photoreceptors and asterisks (*) denote auto-fluorescence in photoreceptor outer segments. (**I**) In situ hybridization for *EGFP* mRNA in a paraffin section from a 4-OHT treated *Tg(krt5:Gal4-ERT-VP16; UAS:EGFP)* animal. The blue arrow shows *EGFP* expression in photoreceptors.

While the zebrafish was developed as a model organism for developmental studies [30], recent years have seen an increased use of zebrafish larvae and adults for behavioral, physiologic, and disease modeling (e.g. cancer) research. Further, given their remarkable ability to regenerate damaged organs, adult zebrafish have become a leading vertebrate model for regeneration studies [31]. Therefore, we investigated the efficacy of the Gal4-ERT system in adult animals. We allowed *Tg(krt5:Gal4-ERT-VP16; UAS:EGFP)* fish to develop to adulthood and induced Gal4-ERT activity by the addition of 1 μM tamoxifen for one hour per day over three days and monitored the fish for EGFP expression. Although the pattern of *krt5* expression in adult zebrafish has not been characterized in detail, we were surprised to observe that, in contrast to developing embryos/larvae, EGFP was not expressed in the epidermis. Rather, tamoxifen-exposed *Tg(krt5:Gal4-ERT-VP16; UAS:EGFP)* adult fish showed restricted EGFP expression in the eye that was drug dependent and increased in intensity following each treatment (Figure 4A–D). Treatment with 1 μM 4-OHT produced similar results (data not shown). A histological analysis of the eyes from tamoxifen-exposed *Tg(krt5:Gal4-ERT-VP16; UAS:EGFP)* fish revealed that EGFP was most notably induced in the photoreceptors, accumulating in the inner segment (Figure 4E–H). By in situ hybridization, *EGFP* transcripts remained present in the photoreceptors eight hours after treatment, which suggests that even transient tamoxifen exposure (only one hour) is sufficient to elicit robust transgene activation in adults with little or no background (Figure 4I). While we were unable to establish if endogenous *krt5* is similarly expressed, the *Tg(krt5:Gal4-ERT-VP16)* line provides a valuable tool for inducible transgene expression in adult zebrafish photoreceptors. More generally, our results validate use of the Gal4-ERT system for spatiotemporal control of transgene expression in adult zebrafish using small molecules.

### Using inducible expression to define temporal roles of Notch signaling

**Figure 5:**
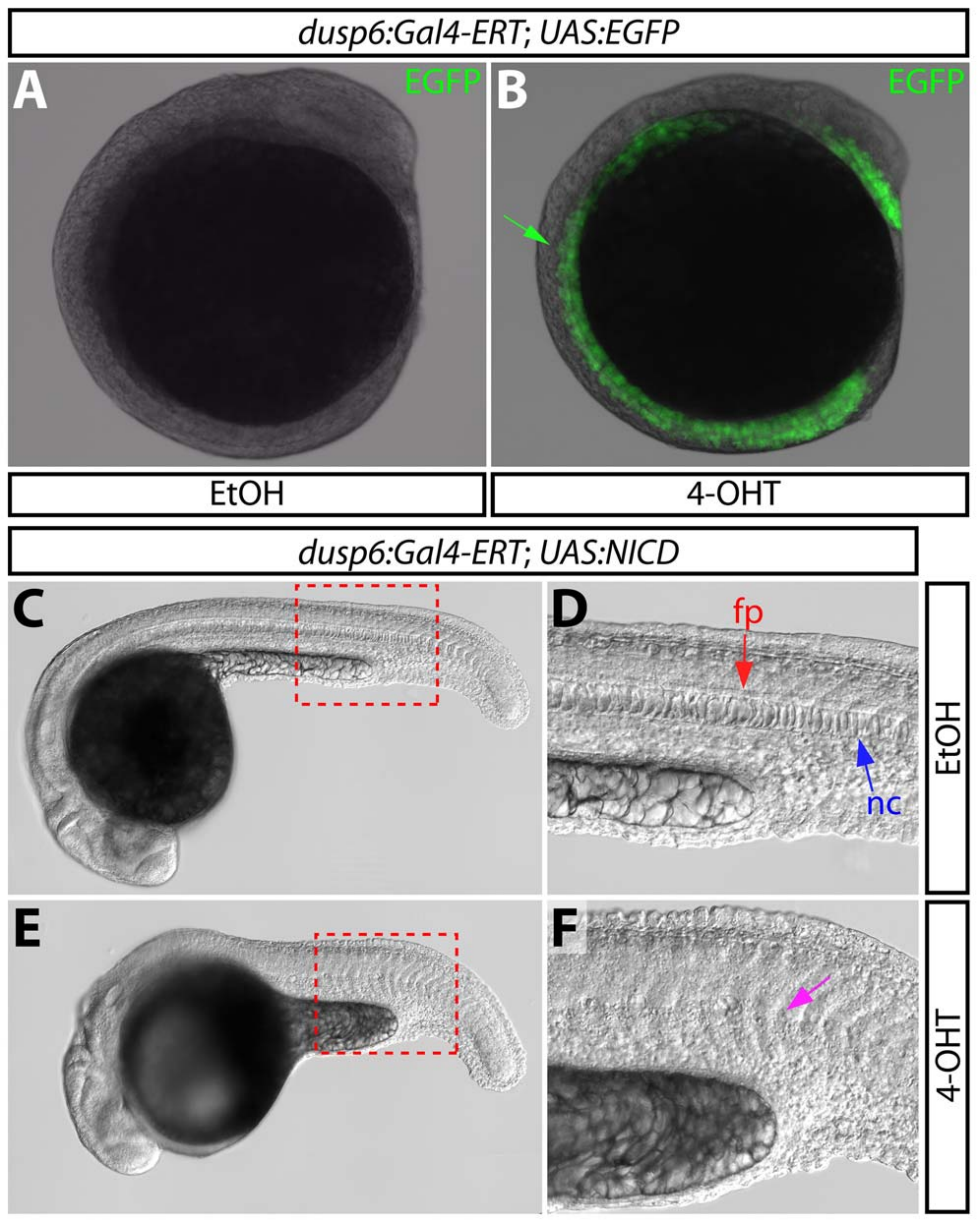
Overexpression of the Notch1 intracellular domain using the *dusp6:Gal4-ERT* driver disrupts notochord development. (**A and B**) EGFP expression at the 10-somite stage in control (A) and 2 μM 4-OHT treated *Tg(dusp6:Gal4-ERT-VP16; UAS:EGFP)* fish. The green arrow points to EGFP expression at the midline. (**C–F**) DIC images of control (C and D) and 4-OHT treated (4 μM from 2–24 hpf, E and F) *Tg(dusp6:Gal4-ERT-VP16; UAS:NICD)* animals at 24 hpf. Regions bounded by the dashed red box in panels C and E are shown in high magnification in panels D and F, respectively. In panel D, the red arrow indicates the floor plate (fp) and the blue arrow indicates the notochord (nc); in panel E, the magenta arrow highlights the reduced notochord and disorganized floor plate.

The high degree of spatiotemporal control over UAS-linked reporters prompted us to ascertain if the system is capable of generating inducible gain-of-function phenotypes. During zebrafish embryogenesis, the organizer forms the notochord, floor plate, and hypochord [32]. Previous studies have established that the Notch signaling pathway within midline precursor cells favors development of the hypochord and floor plate at the expense of the notochord [33], [34]. In support of this model, *Tg(hsp:Gal4; UAS:NICD)* fish ubiquitously overexpressing the Notch1a intracellular domain (NICD) following heat shock [17], [35]–[38], display a dramatically reduced trunk notochord [33]. Given that *Tg(dusp6:Gal4-ERT-VP16; UAS:EGFP)* embryos exposed to 4-OHT from 0–12 hpf exhibited robust EGFP expression in the trunk midline (Figure 5A and 5B), mimicking endogenous *dusp6* [29], we hypothesized expression of activated Notch1a within *dusp6^+^* cells would produce notochord developmental defects. We crossed *Tg*(*dusp6:Gal4-ERT-VP16)* to *Tg(UAS:NICD)* fish and treated resulting embryos with either vehicle or 4 μM 4-OHT beginning at 2 hpf. By 24 hpf, 4-OHT treated embryos were noticeably smaller than controls and had reduced and/or malformed notochords containing misshapen cells (Figure 5C–F), a phenotype reminiscent of that described upon global overexpression of NICD [33], [34]. Such notochord defects were observed in 27% (29/110) of 4-OHT treated embryos at 48 hpf ((Figure S3). As 25% of the embryos would carry both transgenes, the phenotype was fully penetrant. Ethanol treated embryos from the same clutch had no notochord defects (0/100). *Tg*(*krt5:Gal4-ERT; UAS:NICD)* animals expressing NICD in the epidermis following the identical 4-OHT regimen also had normal notochords (Figure S3E–F). These studies show that NICD expression in midline cells is sufficient to disrupt notochord development and validate the Gal4-ERT approach for defining tissue-specific gene function.

**Figure 6:**
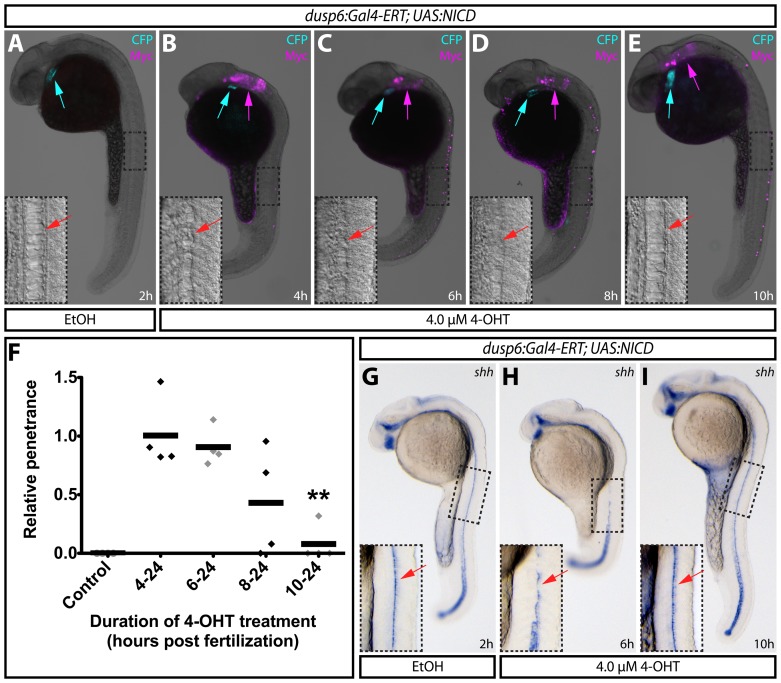
Using inducible expression to define temporal roles of Notch1 signaling in notochord development. (**A–E**) Expression of myc-tagged NICD by immunostaining with myc antibodies on 24 hpf *Tg(dusp6:Gal4-ERT-VP16; UAS:NICD)* embryos treated with ethanol (A) or 4 μM 4-OHT at the indicated times (B–E). Blue arrows indicate myocardial ECFP expression to mark transgenic animals and magenta arrows show expression of myc-tagged NICD. Panel insets display high magnification images of boxed regions where red arrows indicate the notochord (A and E) and the missing notochord (B–D). (**F**) Normalized penetrance of notochord defects in *Tg(dusp6:Gal4-ERT-VP16; UAS:NICD)* animals treated with 4-OHT at the indicated stages of development. Each data point represents the normalized fraction of affected animals in sets of treated embryos from four independent clutches. The data is normalized to the average fraction of abnormal fish in the four 4–24 h sets. The double asterisk indicates a significant difference between 4–24 h and 10–24 h 4-OHT treated fish (P<0.005). (**G–I**) Expression of *shha* in the floor plate of animals treated with ethanol (G) or with 4-OHT for the indicated times (H–I). Boxed regions are shown at higher magnification in the panel insets. Red arrows indicate *shha* expressing floor plate cells.

Lastly, we tested whether the inducible Gal4-ERT system could be used to map the developmental window in which notochord development is sensitive to elevated Notch signaling. We treated embryos with vehicle or 4-OHT at 4 hpf, 6 hpf, 8 hpf, or 10 hpf and scored fish for a notochord reduction phenotype at 24 hpf. The frequency of notochord defects remained constant when embryos were treated with 4-OHT at or before 6 hpf (occurring in approximately 25% of embryos, the expected frequency of double heterozygous animals). In contrast, the number of affected animals decreased when the treatment began at 8 hpf and approached zero when fish were first exposed at 10 hpf (Figure 6A–E, relative penetrance plotted in F). By immunostaining for the myc tag on the NICD transgene, we confirmed that these latter embryos expressed NICD (Figure 6A–E), demonstrating that the lack of a phenotype when 4-OHT was added at 10 hpf was not due to a failure to induce NICD. In situ hybridization for the floor plate and notochord marker *sonic hedgehog* (*shha*) [39], [40] further confirmed that the phenotype was fully suppressed when exposed to drug at 10 hpf in contrast to earlier treatments (Figure 6G–I). These results suggest that Notch activation within midline cells can inhibit notochord formation during gastrulation (between 5 and 10 hpf). After this period, the cells are refractory to elevated levels of Notch signaling. These results are consistent with published data proposing that Notch mediates cell fate decision in the organizer between a notochord and hypochord fate [33], [34], [41]. The tamoxifen-inducible NICD approach we describe could be a useful tool to help determine how Notch mediates this and other cell fate decisions. Generally, this study illustrates how an inducible Gal4-ERT system provides sufficient spatiotemporal control over transgene expression to map narrow windows of cell-type specific gene function.

## Conclusions

Our results validate the use of the Gal4-ERT system in zebrafish by demonstrating how it can direct transgene expression in a cell-type specific and tamoxifen/4-OHT-dependent manner. The approach has substantial advantages over other methods to induce transgene expression in zebrafish, most notably heat shock sensitive promoters. First, the tamoxifen system is dose dependent and therefore transgene expression can be tuned to a desired level by titrating the concentration of tamoxifen/4-OHT added directly to the fish water. Second, at doses that activate Gal4-ERT, tamoxifen/4-OHT causes no adverse effects to zebrafish. In contrast, by definition, heat shock treatment is of substantial stress to zebrafish and may be detrimental to animal health and lead to mixed or obscured results. Finally, heat shock promoters are broadly expressed upon heat shock whereas tissue-specificity is desired or required for many experiments. Tetracycline-inducible transgene expression represents a parallel methodology with similar advantages for zebrafish studies [2], albeit with a more limited number of available Tet-responsive transgenes. Importantly, Gal4-ERT and Tet-On systems are orthogonal and could be used to independently induce expression of two transgenes either simultaneously or sequentially.

Gerety et al. [42] have also demonstrated the feasibility of a Gal4-based tamoxifen inducible transgene system in zebrafish. There are similarities and differences between this report and our study. First, Gerety et al. use a ERT-Gal4 fused to two copies of a truncated VP16 transactivation domain [20] whereas we use Gal4-ERT fused to a single copy of a VP16 [11]. Despite these differences, both inducible Gal4-fusion proteins require similar concentrations of 4-OHT for transgene expression and the kinetics of induction are comparable [42]. Gerety et al. additionally observe the reversal of gene expression following tamoxifen removal. The reversal kinetics may be slow, as we did not see a notable decrease in EGFP expression 24 hours after removing tamoxifen from embryos (data not shown), although the long half-life of EGFP protein complicates this analysis. Using a line (*cldnb:ERT2-Gal4*) directing Gal4-ERT expression primarily in the skin and lateral line, Gerety et al. show the approach can be used to drive tissue-specific expression. We present two additional Gal4-ERT lines (*dusp6:Gal4-ERT* and *krt5:Gal4-ERT*) that provide spatially restricted and tamoxifen-controlled transgene activation. We further validate using the Gal4-ERT system in adult zebrafish. Both reports demonstrate use of the Gal4-ERT system to generate gain-of-function phenotypes. We extend this approach to determine tissue-specific and temporal windows of gene function during development. Collectively, the two reports provide a robust validation of the capability and versatility of the Gal4-ERT system in zebrafish and provide an initial collection of transgenic tools for immediate use.

Potential applications for Gal4-ERT inducible expression transcend the reporter and inducible gain-of-function experiments demonstrated to date. For example, the approach could be used for cell-specific loss-of-function studies by the inducible expression of dominant negative proteins, pathway inhibitors, or shRNAs for RNA interference. Additionally, the inducible expression of a *UAS:Cre* line in concert with floxed reporter lines could improve cell lineage analyses. Lastly, the utility of the system in adults opens up previously intractable experiments in neuroscience, cancer modeling, and organ regeneration research.

## Supporting Information

Figure S1 ***krt5:Gal4-ERT*** **expression matches that of endogenous** ***krt5*** **.** (**A–C**) Whole mount in situ hybridization of *GFP* expression in ethanol (A) or 4-OHT-exposed (B) 24 hpf Tg(*krt5:Gal4-ERT-VP16; UAS:EGFP)* embryos compared to endogenous *krt5* expression (C). Boxed regions are shown at higher magnification in the panel insets.
(TIF)

Figure S2 **Multiple UAS reporter lines produce robust and dose dependent responses to activated Gal4-ERT.** (**A–C**) The *krt5:Gal4-ERT* line is compatible with *UAS:EGFP* (A), *UAS:NICD* shown by anti-myc immunostaining (B), and *UAS:Zebrabow* (C) reporter lines at 36 hpf. (**D–G**) mCherry expression upon treatment of *Tg(krt5:Gal4-ERT-VP16; UAS:Zebrabow)* animals with ethanol or the indicated dose of 4-OHT from 4–36 hpf.
(TIF)

Figure S3 **Tissue-specific notochord defects upon** ***dusp6:Gal4-ERT*** **driven** ***NICD*** **overexpression persist in 48 hpf zebrafish.** (**A–D**) Differenial interference contract images of *Tg(dusp6:Gal4-ERT-VP16; UAS:NICD)* animals treated with either ethanol (A–B) or 4 μM 4-OHT from 2–48 hpf (C–D). Boxed regions in A and C are shown in higher magnification in B and D respectively. Red arrows indicate notochord. Numbers in A and C reflect the quantity of animals displaying a notochord defect in each treated population (25% double transgenic animals). (**E–G**) Bright-field images overlaid with anti-myc immunostaining (magenta) of *Tg(dusp6:Gal4-ERT-VP16; UAS:NICD)* (E–F) and *Tg(krt5:Gal4-ERT-VP16; UAS:NICD)* (G) fish demonstrate that notochord defects are only observed when *UAS:NICD* is driven by *dusp6:Gal4-ERT* (F). Boxed regions are shown in higher magnification in panel insets. Red arrows indicate the notochord (nc).
(TIF)
