# Supplementary figures and images for: Spatial and Temporal Control of Transgene Expression in Zebrafish
Source: PLoS One. 2014 Mar 18;9(3):e92217. doi: 10.1371/journal.pone.0092217 (PMC3958484; doi:10.1371/journal.pone.0092217)

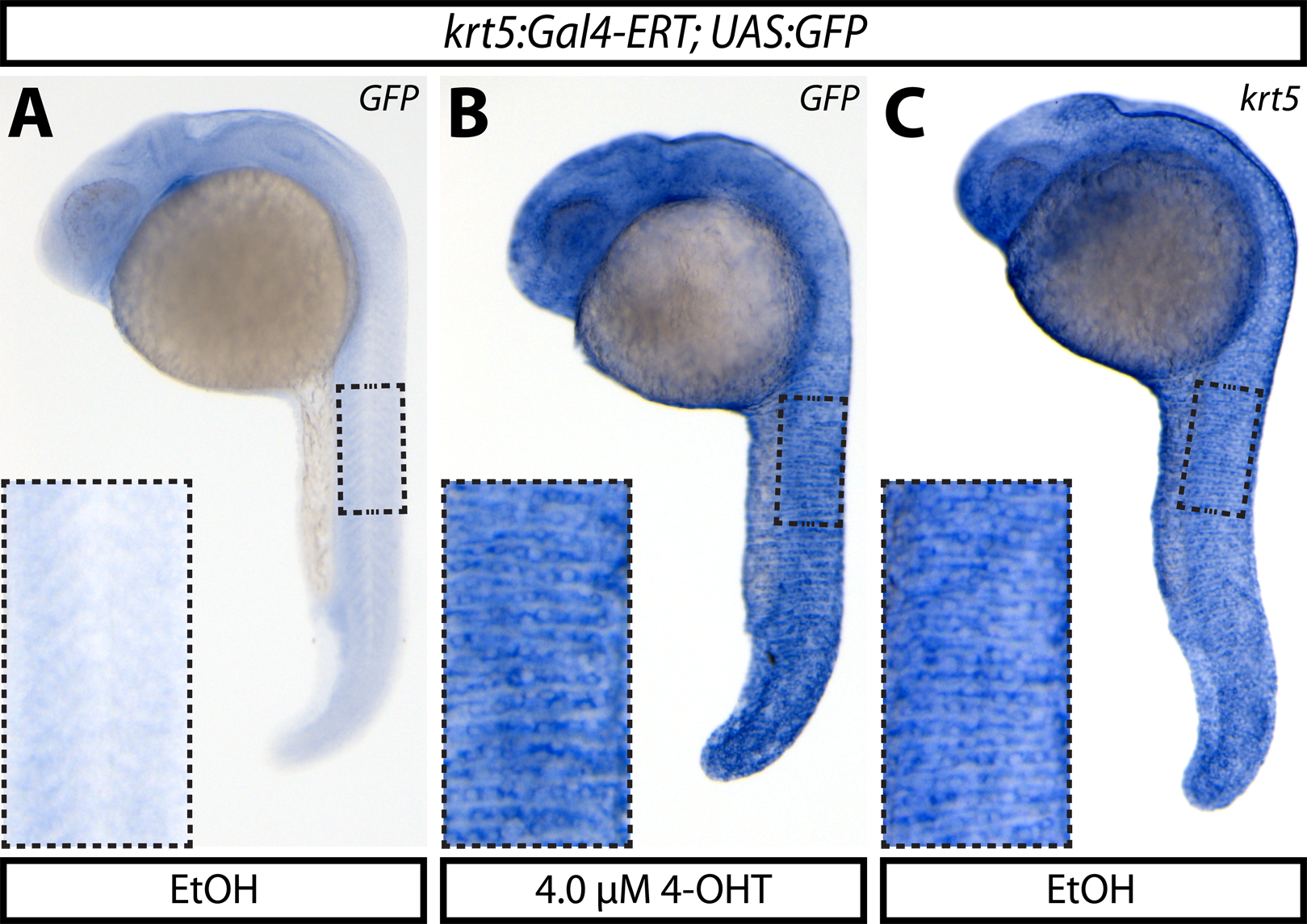

Supplement: Figure S1 — krt5:Gal4-ERT expression matches that of endogenous krt5 . (A–C) Whole mount in situ hybridization of GFP expression in ethanol (A) or 4-OHT-exposed (B) 24 hpf Tg(krt5:Gal4-ERT-VP16; UAS:EGFP) embryos compared to endogenous krt5 expression (C). Boxed regions are shown at higher magnification in the panel insets. (TIF) [file pone.0092217.s001.tif]

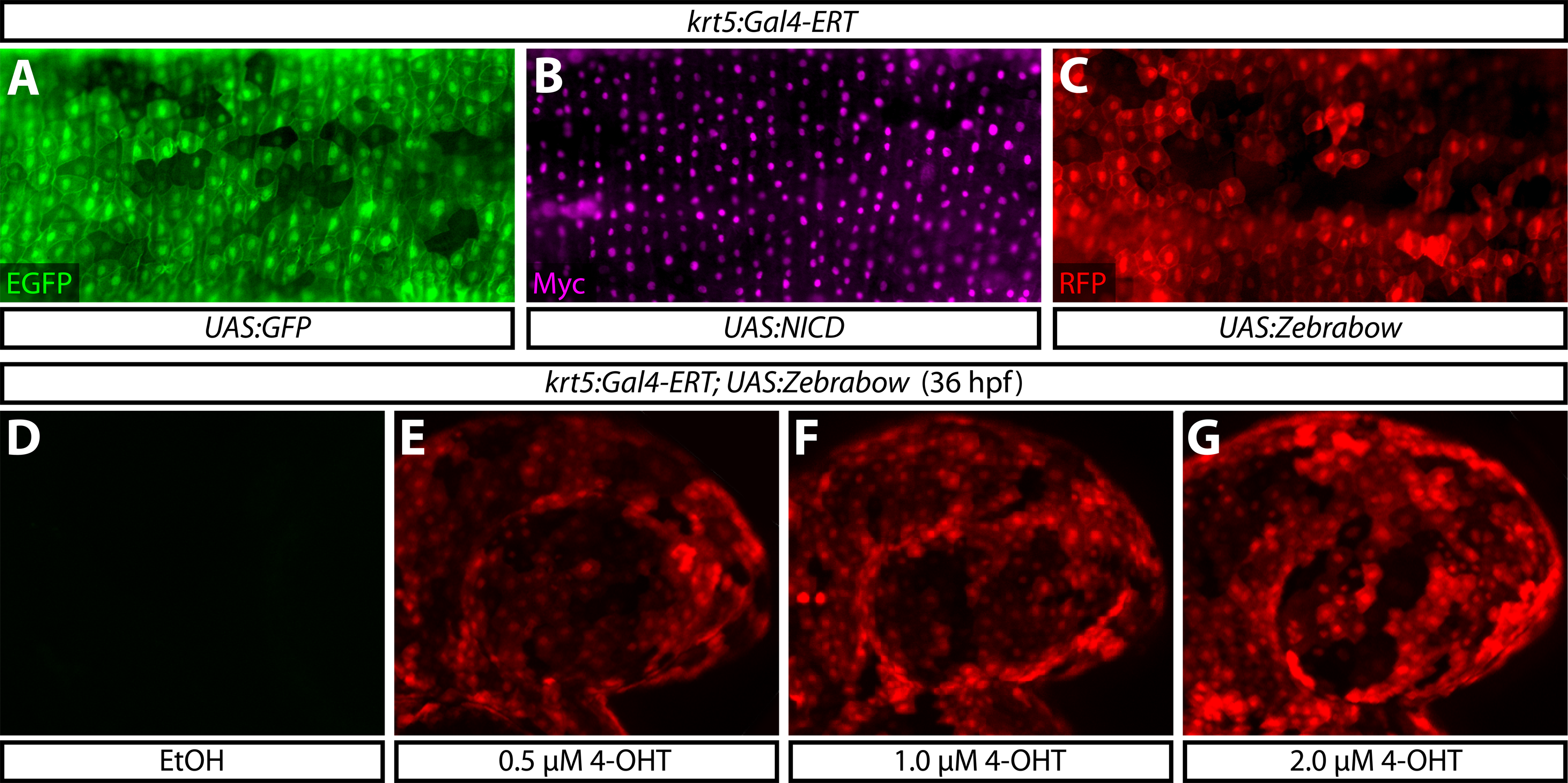

Supplement: Figure S2 — Multiple UAS reporter lines produce robust and dose dependent responses to activated Gal4-ERT. (A–C) The krt5:Gal4-ERT line is compatible with UAS:EGFP (A), UAS:NICD shown by anti-myc immunostaining (B), and UAS:Zebrabow (C) reporter lines at 36 hpf. (D–G) mCherry expression upon treatment of Tg(krt5:Gal4-ERT-VP16; UAS:Zebrabow) animals with ethanol or the indicated dose of 4-OHT from 4–36 hpf. (TIF) [file pone.0092217.s002.tif]

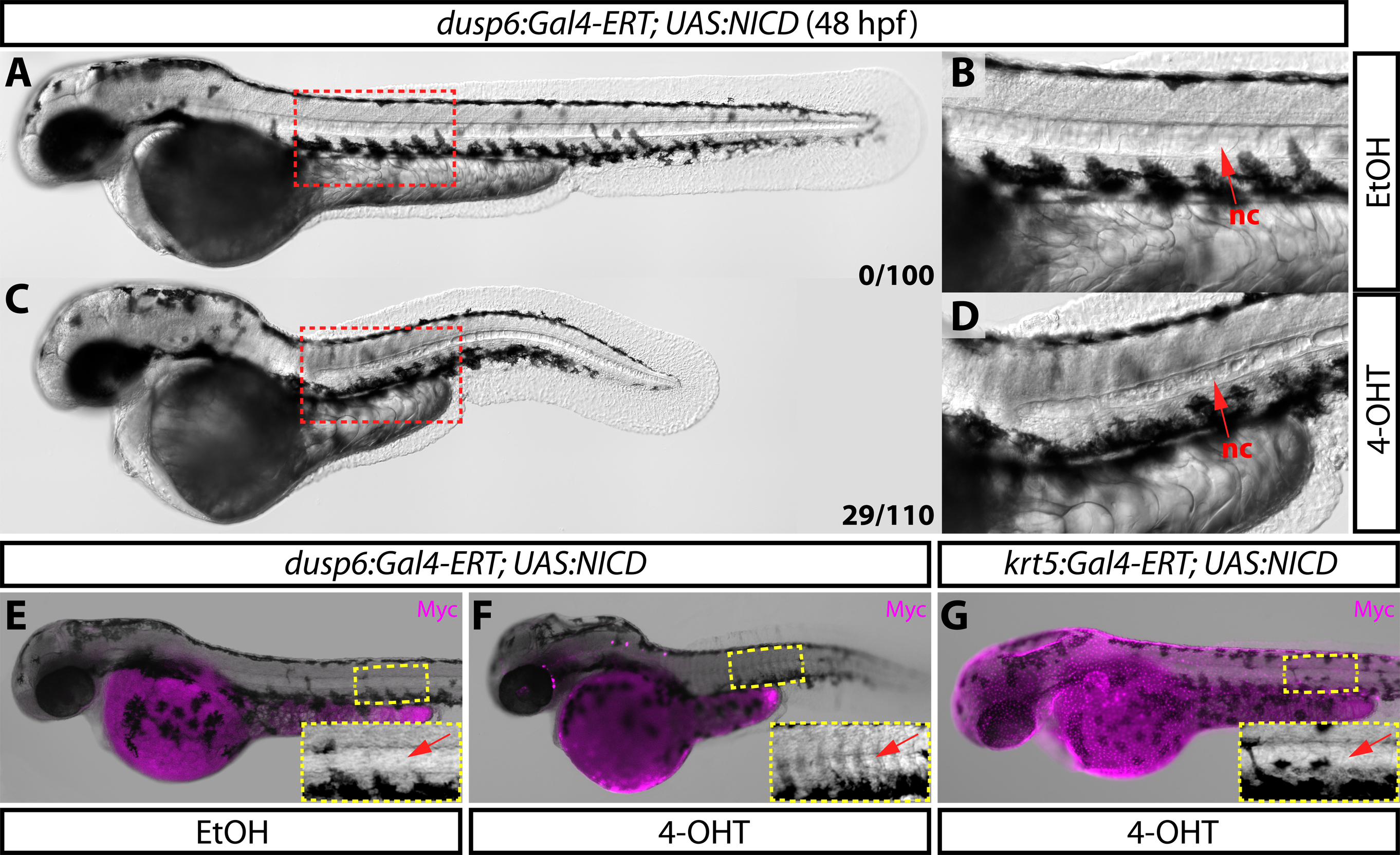

Supplement: Figure S3 — Tissue-specific notochord defects upon dusp6:Gal4-ERT driven NICD overexpression persist in 48 hpf zebrafish. (A–D) Differenial interference contract images of Tg(dusp6:Gal4-ERT-VP16; UAS:NICD) animals treated with either ethanol (A–B) or 4 μM 4-OHT from 2–48 hpf (C–D). Boxed regions in A and C are shown in higher magnification in B and D respectively. Red arrows indicate notochord. Numbers in A and C reflect the quantity of animals displaying a notochord defect in each treated population (25% double transgenic animals). (E–G) Bright-field images overlaid with anti-myc immunostaining (magenta) of Tg(dusp6:Gal4-ERT-VP16; UAS:NICD) (E–F) and Tg(krt5:Gal4-ERT-VP16; UAS:NICD) (G) fish demonstrate that notochord defects are only observed when UAS:NICD is driven by dusp6:Gal4-ERT (F). Boxed regions are shown in higher magnification in panel insets. Red arrows indicate the notochord (nc). (TIF) [file pone.0092217.s003.tif]
